# Void Content, Tensile, Vibration and Acoustic Properties of Kenaf/Bamboo Fiber Reinforced Epoxy Hybrid Composites

**DOI:** 10.3390/ma12132094

**Published:** 2019-06-28

**Authors:** Ahmad Safwan Ismail, Mohammad Jawaid, Jesuarockiam Naveen

**Affiliations:** 1 Laboratory of Bio composite Technology, Institute of Tropical Forestry and Forest Products (INTROP), Universiti Putra Malaysia, UPM Serdang, Selangor 43400, Malaysia; 2 Department of Mechanical and Manufacturing Engineering, Faculty of Engineering, Universiti Putra Malaysia, UPM Serdang, Selangor 43400, Malaysia

**Keywords:** hybrid composites, kenaf, bamboo, acoustic properties, vibration, tensile properties, void content

## Abstract

This study aims to investigate the void content, tensile, vibration and acoustic properties of kenaf/bamboo fiber reinforced epoxy hybrid composites. The composites were made using the hand lay-up method. The weight ratios of kenaf/bamboo were 30:70, 50:50 and 70:30. Further, kenaf and bamboo composites were fabricated for the purpose of comparison. The hybridization of woven kenaf/bamboo reduced the void content. The void contents of hybrid composites were almost similar. An enhancement in elongation at break, tensile strength and modulus of hybrid composites was observed until a kenaf/bamboo ratio of 50:50. Kenaf/bamboo (50:50) hybrid composite displays the highest elongation at break, tensile strength and modulus compared to the other hybrid composites which are 2.42 mm, 55.18 MPa and 5.15 GPa, respectively. On the other hand, the highest natural frequency and damping factors were observed for Bamboo/Kenaf (30:70) hybrid composites. The sound absorption coefficient of composites were measured in two conditions: without air gap and with air gap (10, 20, 30 mm). The sound absorption coefficient for testing without air gap was less than 0.5. Introducing an air gap improved the sound absorption coefficient of all composites. Hence, hybrid kenaf/bamboo composites exhibited less void content, as well as improved tensile, vibration and acoustic properties.

## 1. Introduction

Nowadays, researchers are focused on developing sustainable materials using natural fibers as reinforcement in polymeric composites. There are numerous benefits to using natural fibers as a reinforcement including cost-effectiveness, their non-abrasive nature, low energy consumption, and light weight [1,2]. Moreover, agro wastes such as pineapple leaf and oil palm fruit bunch can be used as a reinforcement in polymeric composites. Besides that, some plants such as kenaf have been cultivated in a large scale to extract its fiber, since this plant was considered as a fast-growing plant in Malaysia. Also, kenaf fiber is readily available in the market and its production can be increased depending on the demand. Kenaf fiber has been widely used as a reinforcement in automobile, aerospace and marine applications due to its higher strength to weight ratio and stiffness [3,4,5,6]. Akhtar et al. studied the mechanical properties of kenaf/ polypropylene composites with different Kenaf fiber loading (0 wt.%, 10 wt.%, 20 wt.%, 30 wt.%, 40 wt.%, 50 wt.%). From the analysis they observed that 40 wt.% of Kenaf fiber loading has shown optimum mechanical properties [7].

The hybridization of natural fiber with another natural fiber or synthetic fiber will combine the advantages of both constituents and result in superior properties. Researchers investigated the influence of hybridizing kenaf and oil palm fiber in the epoxy composites on mechanical properties [8]. In this study, different ratios of oil palm: kenaf were used (70:30, 50:50, 30:70). It was found that hybrid composites with ratio 50:50 exhibited better mechanical properties. Natural fiber attracted researchers to evaluate its capability as a sound insulation material. Lim et al. [9] investigated the sound absorption of kenaf fiber for normal and random sound incidence. It was found that for both methods, the sound absorption coefficient of bulk density 140–150 kg/m^3^ and thickness 25–30 mm at frequency from 500 Hz is more than 0.5 and for frequency above 1.5 Hz, the absorption coefficient is 0.85. In addition, the air gap will improve the absorption towards a lower frequency. Moreover, other researchers have studied the sound absorption of organic multi-layer coir fibers [10]. The finding showed that multi-layer coir has good sound absorption coefficient of 0.85, average from frequency 1000 Hz. Rajesh et al. [11] investigated the free vibration analysis of banana, sisal and banana/sisal hybrid composites. Different weight compositions of fiber (20, 35, 50 and 60 wt.%) were used and they found that a 50% composite showed the maximum natural frequency, while 35% showed the highest damping value.

The purpose of this study is to develop and characterize the properties of woven kenaf/bamboo reinforced epoxy hybrid composites for food tray table application in the automotive industry. Kenaf, bamboo and hybrid composites were fabricated using the hand lay-up method. The tensile, vibration and acoustic properties of composites were evaluated. In addition, the void content of the composites was investigated for the hybrid composites.

## 2. Experimental

### 2.1. Materials

**Figure 1:**
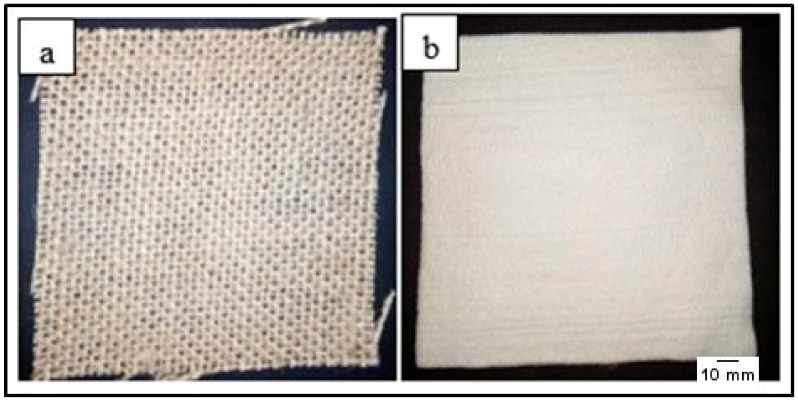
(**a**) Woven kenaf mat (**b**) Bamboo mat.

Woven kenaf mats and bamboo fiber mat were used in this study. Woven kenaf mat is a plain weave while bamboo mat is punched mat. The areal density of each woven kenaf mat and bamboo mat were 600 g/m^2^ and 800 g/m^2^ respectively. The diameter of kenaf fiber and bamboo fiber are 21.9 µm and 11.6 µm respectively. There is no fiber treatment for both mats. Woven kenaf mat and bamboo mat, as shown in Figure 1, were procured from Zul Sdn. Bhd, Selangor, Malaysia and Shijiangzhung Bi Yang Technology Co. Ltd., Hebei, China. The epoxy resin, epoxy hardener and silicon spray were supplied by Tazdiq Engineering Sdn. Bhd, Selangor, Malaysia. Table 1 and Table 2 shows the properties of epoxy resins (D.E.R * 331) and joint amine hardener (905-3S).

### 2.2. Fabrication of Composites

A stainless steel mould with dimensions of 300 mm × 300 mm × 5 mm were made and woven kenaf and bamboo mat were cut according to mould size. Bamboo mat, woven kenaf and hybrid woven kenaf/bamboo reinforced hybrid composites were fabricated using the hand lay-up method. Hybrid composites of woven kenaf and bamboo mat were prepared with different weight ratios such as 70:30, 50:50 and 30:70 with a total fiber/matrix weight ratio as 40/60. An epoxy and hardener were mixed with a 2:1 ratio and stirred for about 2–4 min. Initially, the epoxy was poured into the mould followed by bamboo mat and woven kenaf were placed inside the mould. Epoxy was applied on every layer of Kenaf and bamboo mats. Every composite was made using four-layer mat. Then, the mould was placed in a hot press at 110 °C for 10 min followed by a cold press for 5 min. Single woven kenaf mat and bamboo mats were prepared as control. Sample codes are listed in Table 3.

### 2.3. Characterization

#### 2.3.1. Void Content

Voids in hybrid composites were determined as per ASTM-D-2734-70. The void content was calculated by using following Equations (1)–(4).

r = M_F_/M_B_ × 100 (1)

R= 100 − r(2)

T_d_ = 100/(R/D + r/d) (3)

Void Content = 100(T_d_**−** M_d_)/T_d_ (4)

where, R is the weight % of the resin in the composite, r is the weight % of the reinforcement in the composite; M_F_ is the mass of fiber, M_B_ is mass of composite, D is the density of the resin matrix, d is the density of the reinforcement, T_d_ is theoretical density and M_d_ is measured density.

#### 2.3.2. Tensile Testing

The specimen for tensile testing was prepared with a dimension of 120 mm × 20 mm × 5 mm by using band saw. The samples were tested according to the ASTM D3039 using 30 kN Bluehill INSTRON 5567 universal Testing machine (Instron, Shakopee, USA). The gauge length used was 60 mm and testing speed was set at 5 mm/min. The samples were put in conditioning chamber for a day with temperature 23 ± 3 °C and relative humidity 50 ± 10%. Five replicates were tested in each sample and the average values were reported.

#### 2.3.3. Scanning Electron Microscopy (SEM)

The surface morphology of the fracture surface of the tensile samples was examined by using Hitachi S-3400N scanning electron microscopy (SEM) (Hitachi, Krefeld, Germany) with an acceleration voltage of 10 kV. The samples were coated with a thin layer of gold prior to morphological analysis in order to obtain clear images.

#### 2.3.4. Modal Analysis (Free Vibration Test)

**Figure 2:**
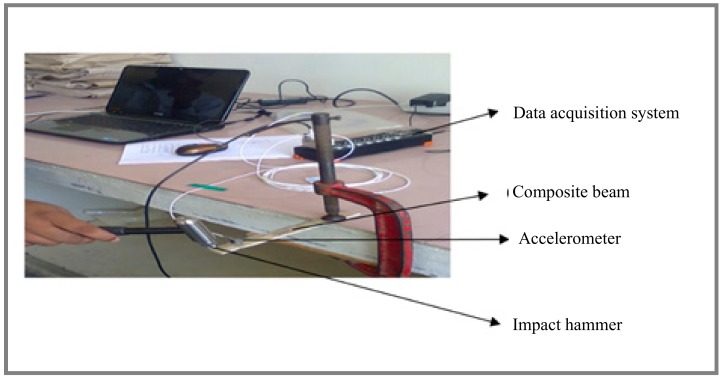
Experimental setup for free vibration test.

Modal analysis was employed to study the dynamic characteristics (natural frequency and damping behavior) of the composite beams. In this study, an impact hammer (Kistler model 9722A500) was used to perform the modal analysis as shown in Figure 2. The dimension of the composite beam is 200 mm × 20 mm × 3 mm. A cantilever beam was used to perform modal analysis. The free end of the composite beam was attached to an accelerometer. The signals from the accelerometer were recorded with a DAS (data acquisition system) and ICP (Integrated Circuit Piezoelectric) connector which were connected to a personal computer. The output signals were captured using two separate adaptors (i.e.,) one for accelerometer and another for impact hammer. In the free vibration test, the response of the beam after hitting the beam with an impact hammer was tracked and the computed FRF (frequency response function) provides information regarding the natural frequency of the composite beam. The damping factor of the hybrid composite beam was calculated using a half-power band according to the method. The expression for damping factor (ζ) is given by Equation (5).

(5) $$\zeta =\frac{\Delta \omega }{2\omega_{n}}$$

where, Δω represents the bandwidth of the resonance peak; ω_n_ indicates the fundamental natural frequency.

#### 2.3.5. Acoustic Properties

**Figure 3:**
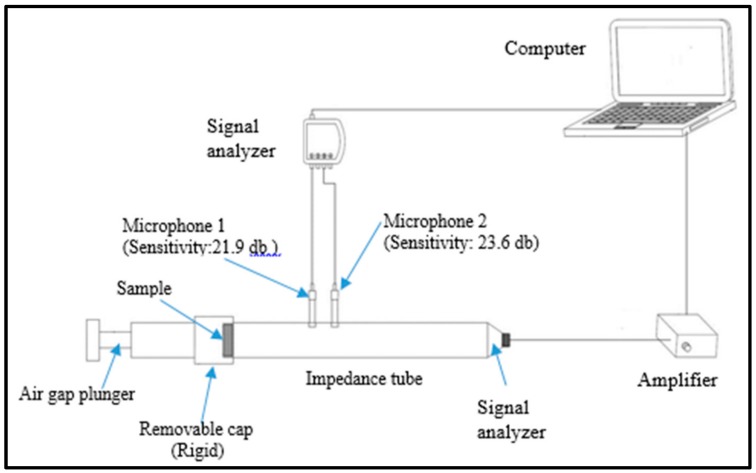
Experiment set up for acoustic testing.

**Figure 4:**
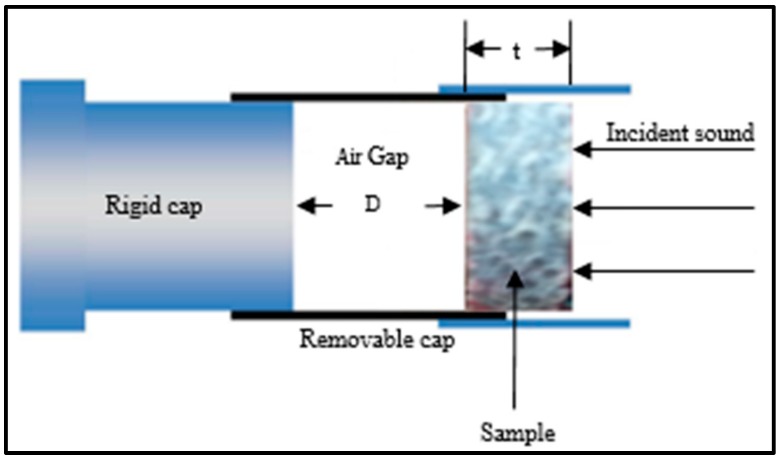
Arrangement of specimen inside the removable cap in the impedance tube.

Normal-incident sound absorption coefficient of hybrid woven kenaf/bamboo mat, woven kenaf and bamboo mat composite were measured using impendence tube based on the transfer function method according to ISO 10534-2. The diameter of the sample is 33 mm. Figure 3 shows the experiment set up for acoustic testing. The internal diameter of tube is 33.3 mm and external diameter is 43 mm. The distance between sample and loudspeaker is 320 mm. The specimens were positioned inside the removable cap and placed at one end of the impedance tube (Figure 4) which is opposite the loudspeaker. After placing the sample, white noise was fed into the tube to provide equal sound energy per constant bandwidth/Hz. The incident sound from sound source and the reflected sound from the sample were recorded by the two acoustic microphones (RION U 57) in front of the sample. The signal was recorded by an analyzer after 10 min of sending white noise and the data were processed in the computer to generate the auto and cross-spectra required to produce the transfer function. The absorption coefficient was calculated based on the measurement transfer function. The sound absorption coefficient will be generated based on the effective frequency range (500 Hz to 4500 Hz). The effect of various air gaps (0 mm, 10 mm, 20 mm, and 30 mm) on the sound absorption coefficient of the composites was evaluated.

## 3. Results and Discussion

### 3.1. Void Content

The void content will reduce the physical and mechanical properties of the composites. Table 4 shows the void content of the composites. K shows the highest amount of void content which is 7.56% and lowest in 3B7K hybrid composites (3.20%). The presence of void content is due to the inefficiency of the polymeric phase to displace the trapped air within the composites [12]. Besides that, an increase in void also occurred due to the incomplete wetting out of fibers by the matrix [13,14]. In addition, the formation of void content was also due to the presence of moisture in the fiber during fabrication. There are hollow structures in kenaf fiber, the resin cannot penetrate into the hollow structure of fibers. Besides that, void might occur during the resin preparation of epoxy resin and hardener due to air entrapment. Jawaid et al. studied the void content of empty fruit bunch (EFB)/jute reinforced epoxy hybrid composites [15]. It was reported that the porous nature of EFB fiber was one of the reasons for the higher void content.

### 3.2. Tensile Properties

**Figure 5:**
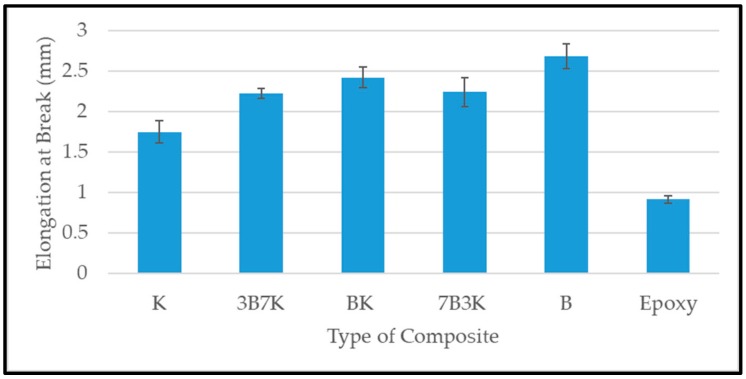
Elongation at break of woven kenaf, bamboo mat and woven kenaf/bamboo mat hybrid composite with different ratios.

Elongation at break indicates the damage tolerance of composites [12]. A higher elongation at break of a composite means that it has better damage tolerance. The elongation at break of the K, B, and hybrid composites were shown in Figure 5. B has the highest elongation at break, while K has lowest. The hybridization of kenaf with bamboo improved the elongation at break of the composites. An increase in the ratio of bamboo fiber up to a ratio 50:50 shows an increasing trend in elongation at break of hybrid composites. A further increase in bamboo fiber decreases the elongation at break. This might be due to the failure of kenaf fiber which led 7B3K composite to has lower elongation at break compared to hybrid composite BK. However, 7B3K has a slightly higher in elongation at break compared to 3B7K due to the bamboo fiber which has higher elongation at break compared to kenaf fiber.

**Figure 6:**
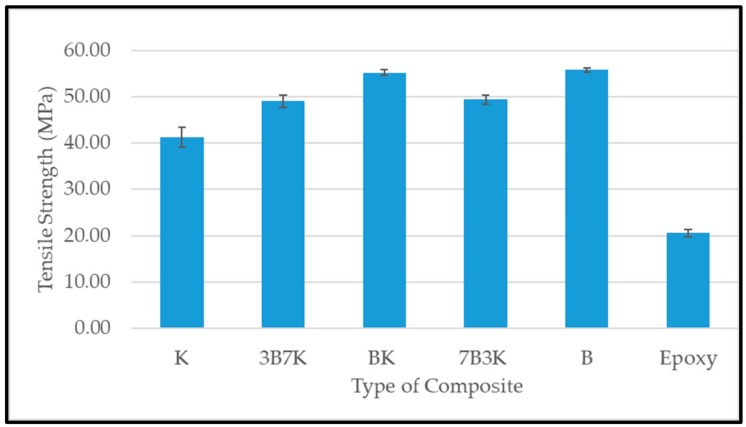
Tensile strength of woven kenaf, bamboo mat and woven kenaf/bamboo mat hybrid composite with different ratio.

**Figure 7:**
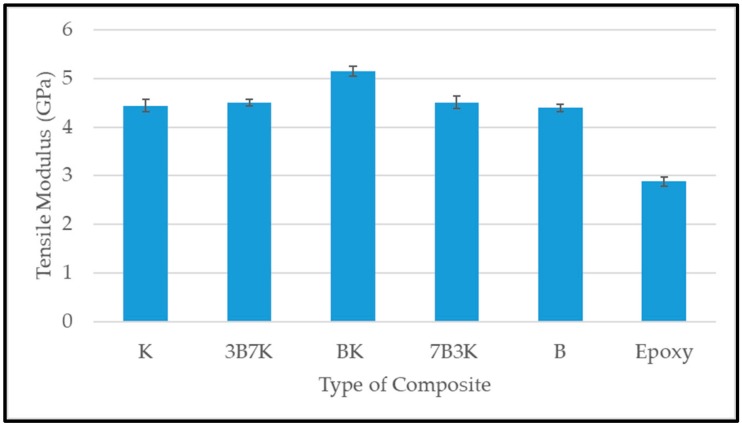
Tensile modulus of kenaf, bamboo and kenaf/bamboo hybrid composite with different ratio.

Figure 6 and Figure 7 shows the tensile strength and modulus of woven kenaf, bamboo mat and woven kenaf/bamboo mat hybrid composites. Several factors influence the tensile properties of composite material. The strength and modulus of the fiber plays a vital role on the tensile properties of fiber reinforced polymer composites [12]. The hybridization of kenaf fiber with bamboo fiber improved tensile properties. An enhancement in tensile properties of hybrid composite was due the advantage of bamboo fiber which is stronger than the kenaf fiber. The elongation at break of bamboo is higher compared to kenaf. According to Zweben et al. [16] the hybridization of high elongation fibers with low elongation fibers in the polymeric composites will enhance the strain level required to propagate the fiber breakage, because high elongation fiber will act as crack arrestors on a micromechanical level. Among the hybrid composites, the hybrid with a ratio 50:50 of kenaf and bamboo showed the highest tensile strength.

Among all the composites, BK has the highest tensile modulus. Even though 7B3K has higher bamboo content compared to BK, it has lower tensile strength and modulus compared BK (55.28 MPa and 5.15 GPa). Lower tensile strength and modulus of 7B3K compared to BK might be due to the failure of kenaf fiber to withstand the applied load on the composite. The individual fiber properties in hybrid composites, such as strength, modulus and elongation at break, will determine the overall performance of the composite [16,17]. Other studies also found that the hybrid composite with a ratio of 50:50 has optimum tensile properties when compared with other hybrid ratios [18,19,20].

### 3.3. Scanning Electron Microscopy (SEM)

**Figure 8:**
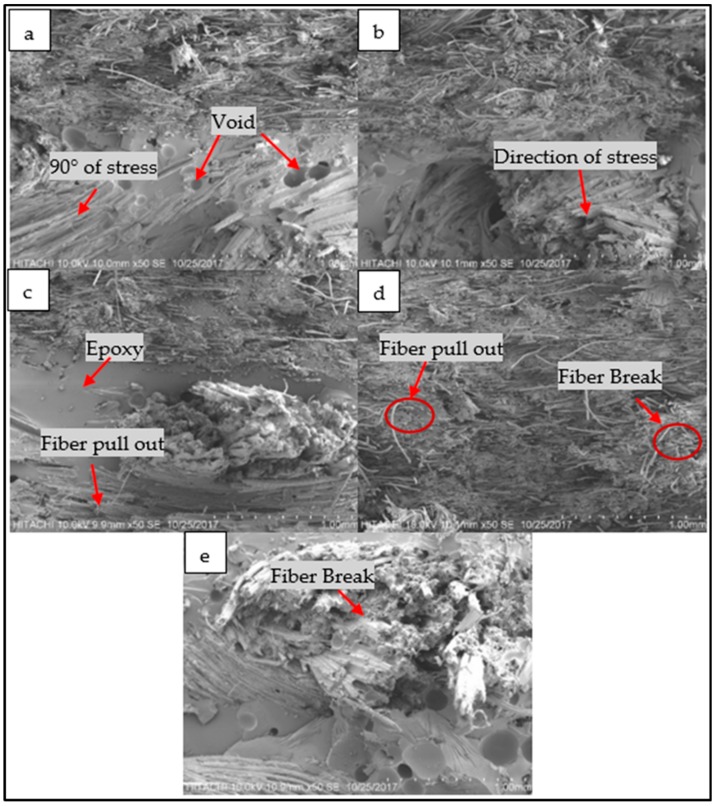
SEM micrograph of tensile fractured composites; (**a**) 7B3K, (**b**) BK, (**c**) 3B7K, (**d**) B, (**e**) K.

Scanning electron microscopy of tensile fractured woven kenaf, bamboo mat and woven kenaf/bamboo mat hybrid composites were shown in Figure 8. The layers of kenaf and bamboo can be seen in the upper part to consist of bamboo fibers and the lower part consists of kenaf fibers (Figure 8a–c). From Figure 8, the voids in the composites might affect the tensile strength and modulus of composites. The presence of a void will decrease the tensile strength and modulus of composites. On the other hand, the hybrid composites exhibited better fiber/matrix adhesion. It can be validated with absence of voids in the hybrid composites. Fiber pull-out and fiber breakage can be seen in all samples. In kenaf part, it can be seen that fiber which positioned at 90° of stress are pulled out compared to the direction of the stress (Figure 8a–c). Lower tensile properties are due to the inefficiency of stress transfer rate to the fiber [21].

**Figure 9:**
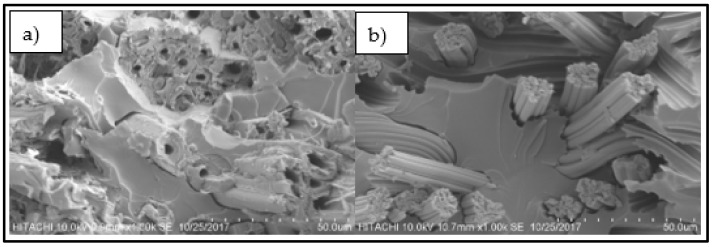
Fiber in composites (**a**) kenaf fiber, (**b**) bamboo fiber.

Figure 9 shows kenaf fiber and bamboo fiber in composites. There are hollow structures in kenaf fiber while it was no hollow structures within bamboo fiber. This hollow structure will contribute to the higher amount of void content in composites and decrease the mechanical properties of composites. This structure has led to a higher amount of void in the K composite compared to others. Void formation during fabrication and the structure of kenaf fiber could be the reason for a lower tensile strength of K compared to other four composites. In acoustic applications, this structure will improve the sound absorption coefficient of composites.

### 3.4. Free Vibration

The natural frequency of natural fiber-based composites depends on factors which include chemical composition, fiber orientation, layering sequence, fiber length and diameter, and fiber/matrix interfacial bonding [22]. The initial three modes of K, B and hybrid kenaf/bamboo (BK, 3B7K, 7B3K) composites were analysed using a simple cantilever beam with impact hammer. The three modes of the composites are as follows: Mode 1 (Bending), Mode 2 (twisting), Mode 3 (second bending). Table 5 displays the natural frequency of the composites at different modes. The hybrid composite (3B7K) exhibited a higher natural frequency among the composites in Mode 1. Further, the hybrid composites (BK, 3B7K, and 7B3K) displayed better natural frequencies compared to K and B composites. From the analysis it was observed that the optimal hybrid kenaf/bamboo was 30/70. Moreover, it showed 18.5% improved natural frequency compared to K and B composites. The increase in natural frequency is attributed to the improved stiffness of the hybrid composites [23]. Mode 2 and Mode 3 also followed the similar trend. Senthil et al. [24] also found that hybrid banana/coconut sheath possessed higher natural frequencies compared to individual banana and coconut sheath composites.

Table 6 presents the damping factor of K, B and hybrid kenaf/bamboo (BK, 3B7K, 7B3K) composites. Hybrid composites (3B7K) exhibited higher damping factor among the composite beams due to higher interfacial interactions between the fiber/matrix and stiffness. Moreover, it showed 54.9% and 265% higher damping value compared to K and B-based composites respectively. Hence hybrid composites, at this optimum weight percentage (Bamboo: 30 wt.%, Kenaf: 70 wt.%) (3B7K) proved that hybrid materials will combine the advantages of their individual constituents and result in superior properties which cannot be obtained from them [25]. Mode 2 and Mode 3 also have shown similar trend. Therefore, it validates the fact that a higher mode shape depends on the fundamental mode shape of the composite structure [24].

### 3.5. Acoustic Properties

**Figure 10:**
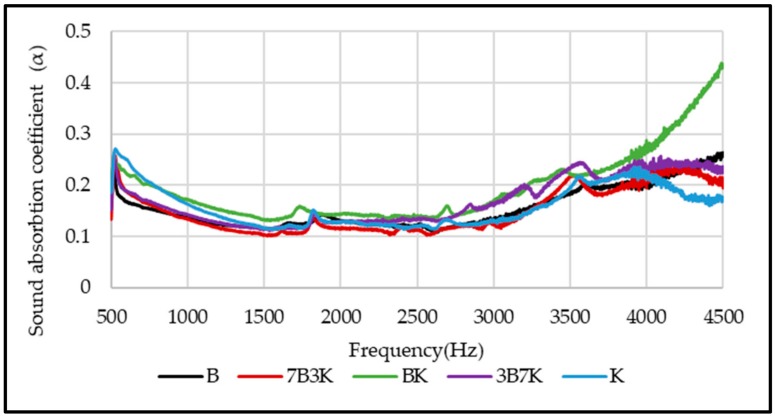
Sound absorption coefficient of woven kenaf, bamboo mat and woven kenaf/bamboo mat hybrid composite with different ratio.

The sound absorption coefficients of woven kenaf, bamboo mat and woven kenaf/bamboo mat hybrid composites are illustrated in Figure 10. The sound absorption coefficient composites showed an almost similar trend, and the values are quite consistent. The graph shows that sound absorption of composites decreases after 500 Hz up to 1500 Hz, while absorption was relatively constant at frequency 1500 Hz to 3000 Hz. The sound absorption coefficient shows increase in sound absorption after frequency 3000 Hz. Sound absorption coefficient of kenaf composites is higher than bamboo composite at frequency between 500 to 1500 Hz. The reason for higher sound absorption of Kenaf composites compared to bamboo mat composite is due to the hollow structure or higher porosity of Kenaf fiber. This hollow structure will trap and absorb the sound that entered inside the composite [26]. The hollow structure of kenaf fiber can be seen in Figure 9. Yang and Li studied the sound absorption coefficient of different natural fiber reinforced epoxy composites [27]. Based on this study, it was found that the sound absorption coefficient was less than 0.5 for the frequency of 0 Hz to 4500 Hz. While comparing the sound absorption of epoxy with natural fiber and without natural fiber, it was found that natural fiber reinforced epoxy has better sound absorption. Among hybrid composites, BK has a slightly higher sound absorption coefficient. 

**Figure 11:**
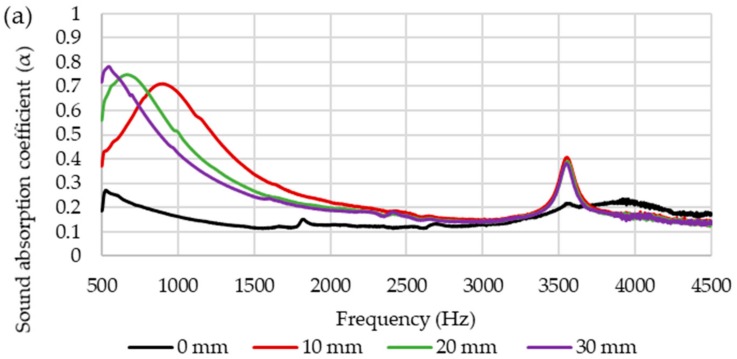

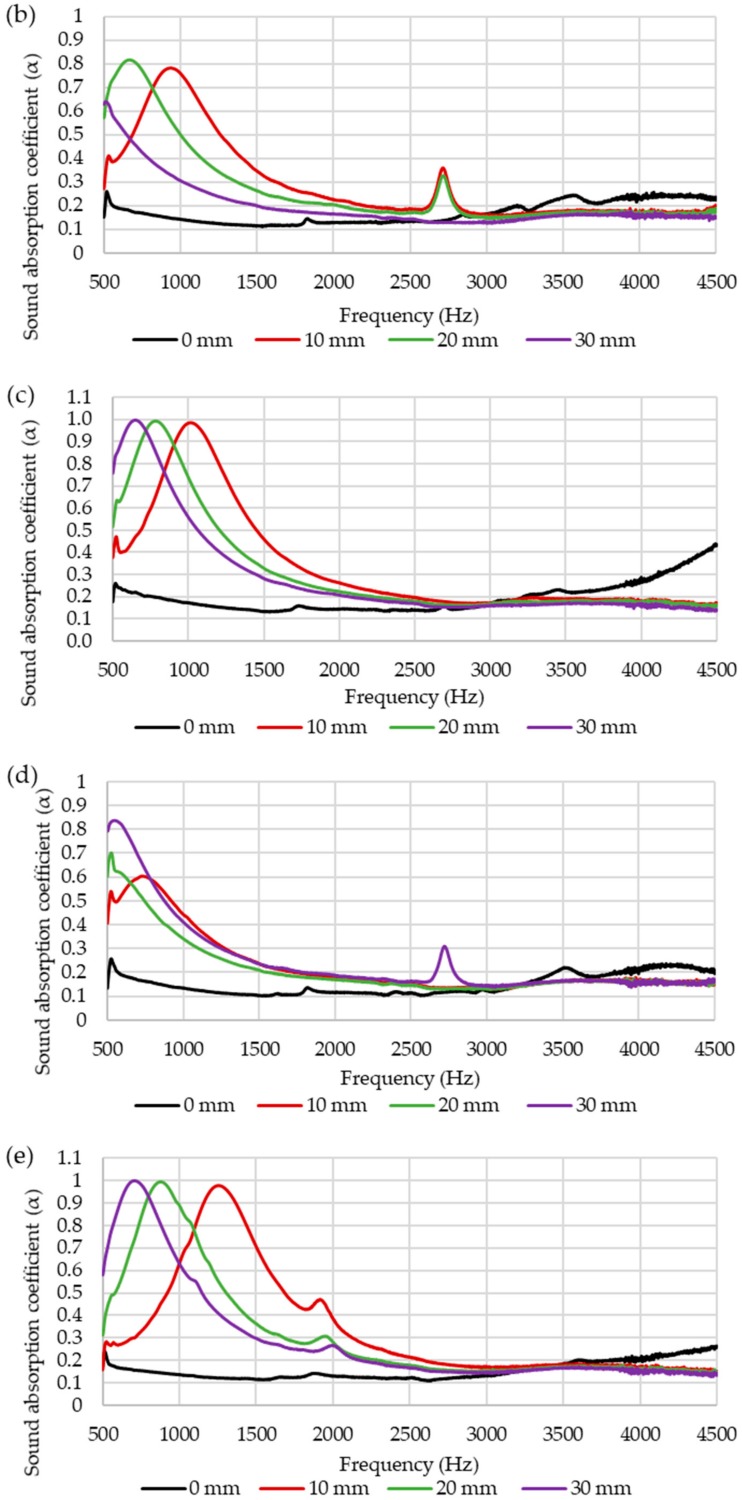
Sound absorption coefficient of composites with different thickness of air gap (**a**) K (**b**) 3B7K (**c**) BK (**d**) 7B3K (**e**) B.

Based on the results, it was observed that the sound absorption of the composites is lower than 0.5 which indicates that the composites absorb sounds less efficiently. The sound absorption of the composite depends on several factors such as air flow resistivity, porosity, viscoelasticity, density, thickness and tortuosity [28]. An air gap between sample and rigid wall of the composites could be introduced to enhance the sound absorption of the composites [9]. Figure 11 displays the effect of different thickness of air gap on sound absorption coefficient of composites. Figure 11a,e shows the influence of air gap on the sound absorption of kenaf and bamboo composites. Sound absorption of kenaf and bamboo composites increased at a frequency range of 500 to 3000 Hz. The peak of maximum sound absorption increased and shifted to a lower frequency as the thickness of the air gap increased. Figure 11b–d shows the effect of air gap on the sound absorption of hybrid composites. Among the hybrid composites, hybrid composites with ratio 50:50 (kenaf/bamboo) displayed better sound absorption with maximum peak sound absorption higher than other two hybrid composites. As the thickness of the air gap increases, the maximum peak sound absorption of hybrid composites shifted to lower frequency. Increasing thickness of the air gap will shift the maximum value of sound absorption from a higher to lower frequency range [9,29].

## 4. Conclusions

The hybridization of woven kenaf and bamboo showed an improvement in overall properties. The analysis of void content showed that there was not much difference in the void content of hybrid composites, but there was a slight improvement compared to the woven kenaf composite. It was demonstrated that the hybrid composite with a ratio of 50:50 showed the highest tensile strength and modulus. Hybridization of kenaf fiber with bamboo has improved the natural frequency compared to woven kenaf and bamboo composites. Of all the composites, 3B7K exhibited the highest natural frequency. In addition, 3B7K had the highest damping factor compared to other composites. The sound absorption coefficient of the composites did not show significant difference. Based on the analysis, hybrid composites with ratio 50:50 of kenaf to bamboo were slightly better in terms of their sound absorption coefficient compared to other composites in the study of sound absorption of composites without an air gap. When an air gap was introduced during the experiment, the sound absorption coefficient of composites improved at a lower frequency. As the thickness of the air gap increased, the maximum peak of sound absorption shifted to lower frequency. We suggest the use of kenaf/bamboo hybrid composites for non-load bearing structures where noise control and sound absorbing properties are prime requirements.

## Figures and Tables

**Table 1:** Properties of epoxy resin.

Property	Value
Epoxide Equivalent Weight (g/eq)	182–192
Epoxide Percentage (%)	22.4–23.6
Epoxide Group Content (mmol/kg)	5200–5500
Color (Platinum Cobalt)	75 Max.
Viscosity @ 25 °C (mPa·s)	11,000–14,000
Hydrolyzable Chloride Content (ppm)	500 Max.
Water Content (ppm)	700 Max.
Density @ 25 °C (g/mL)	1.16
Epichlorohydrin Content (ppm)	5 Max.
Shelf Life (Months)	24

**Table 2:** Properties of hardener.

Property	Value
Amine value (mg KOH/g)	300 ± 20
Viscosity (BH type @ 25 °C, cPs)	200–400
Color (Gardner)	<2
Equivalent Wt (H)	95
Pot life (100 g @ 25 °C)	75 min
Hardness (Shore D)	85
Thin film set time (@ 25 °C)	5 h

**Table 3:** Sample codes.

Code	Density g/cm^3^	Ratio of Kenaf to Bamboo (Kenaf/Bamboo)	
		Kenaf (K)	Bamboo (B)
K	1.0750	100	0
3B7K	1.1475	70	30
BK	1.1450	50	50
7B3K	1.1525	30	70
B	1.1825	0	100

**Table 4:** Void content of composite.

Type of Composites	Void Content (%)
K	7.56
7B3K	4.74
BK	5.59
3B7K	3.20
B	4.37

**Table 5:** Natural frequency of the composites.

Sample Name	Natural Frequency (Hz)		
	Mode 1	Mode 2	Mode 3
K	65.92	131.84	283.2
B	65.92	158.69	319.82
BK	68.3	153.81	327.15
3B7K	78.13	456.54	588.38
7B3K	75.68	456.54	585.94

**Table 6:** Damping factor of the composite.

Sample Name	Damping Factor		
	Mode 1	Mode 2	Mode 3
K	0.1841	0.0266	0.0206
B	0.0781	0.0113	0.0088
BK	0.0791	0.0410	0.0191
3B7K	0.2853	0.1360	0.0675
7B3K	0.0928	0.0471	0.0222
